# Associations of FoxP3 gene polymorphisms with severe recurrent respiratory papillomatosis in Korean patients

**DOI:** 10.1186/s40463-017-0197-z

**Published:** 2017-03-15

**Authors:** Tack-Kyun Kwon, Eun Jae Chung, Nuri Lee, Eun Youn Roh, Eun Young Song

**Affiliations:** 1 Department of Otolaryngology-Head and Neck Surgery, Seoul National University College of Medicine, Seoul, South Korea; 2 Department of Laboratory Medicine, Seoul National University College of Medicine, 101 Daehak-ro, Jongno-gu, Seoul, 03080 South Korea

**Keywords:** Disease susceptibility, Foxp3, Human papilloma virus, Recurrent respiratory papillomatosis, Single nucleotide polymorphism

## Abstract

**Background:**

FoxP3 is the most dependable marker for regulatory T cells which play a major role in immune tolerance. Foxp3 gene polymorphisms were associated with various autoimmune diseases and clearance of viral infections. We studied the association of Foxp3 polymorphisms in severe RRP patients.

**Methods:**

A total of 30 Korean severe RRP patients and 195 healthy controls were enrolled. Foxp3 polymorphisms (rs5902434 del/ATT, rs3761548 C/A, rs3761549 C/T, and rs2232365 G/A) were determined by PCR and sequencing.

**Results:**

Genotype frequencies (GF) of rs5902434 ATT/ATT and rs2232365 GG were significantly decreased in female RRP patients than controls (0.0% vs 23.0%, *p* = 0.039, OR = 9.4 for both).

**Conclusions:**

We showed that Foxp3 polymorphism of rs5902434 and rs2232365 could be an important protective factor in the susceptibility of severe RRP in female Koreans. Further studies on larger number of patients and other ethnic groups are needed to clarify the association.

## Background

Regulatory T (Treg) cells are involved in diseases characterized by dysregulated peripheral tolerance, such as autoimmune diseases and viral reactivation. Forkhead box P3 (FoxP3) is a transcription factor that regulates Treg development and function, and is still the most reliable marker for Treg [1].

Recurrent respiratory papillomatosis (RRP) is a rare benign neoplasm of the larynx and trachea characterized by frequent recurrences of papilloma of the airway with significant morbidity. Although it is caused by human papillomavirus (HPV) types 6 and 11, it is unclear why only a very small fraction of HPV-exposed subjects develop RRP [2]. In RRP patients, complete eradication of HPV is rare, which implicates defects in the host cell-mediated immune response [3]. Recently, the increase of Treg in papilloma tissue but exhaustion and chronic activation of Treg have been reported [4]. *Foxp3* gene polymorphisms in promoter region could affect the function and quantity of Foxp3 molecule, which results in defects in Treg function, have been associated with various autoimmune disease and clearance of viral infections [5, 6]. In this study, we analyzed the association of four Foxp3 polymorphisms (rs5902434 del/ATT, rs3761548 C/A, rs3761549 C/T, and rs2232365 G/A) in promoter region in Korean severe RRP patients.

## Methods

### RRP patients and control subjects

A total of 30 patients (26 adults and 4 children) who underwent surgery for severe RRP in the period from Feb 2010 to Dec 2010 at Seoul National University Hospital were included in this study. The mean age of patients was 41.2 years (4–80 years). Clinical severity was determined by the extent of disease at the time of surgery and the frequency of recurrence as previously described [7]. At each surgery, the clinical and anatomical scores were documented to yield a total score according to previously suggested staging system [8]. The mean growth rate of multiple surgeries was used to define the overall severity score for an individual patient. An overall disease severity score of ≥ 0.06, or more than three surgeries in the previous 12 months, or tracheal extension, was defined as severe RRP [7]. Out of 15 female RRP patients, four patients had medical history of immune-related disorder; Pityriasis lichenoides chronica, Immune chronic urticaria, Erysipelas, and allergic reaction to various drugs such as NSAIDs, cefazolin, and radiocontrast agent, respectively. Out of 15 male RRP patients, one patient had allergic history to radiocontrast agent. Ethylenediamine tetraacetic acid (EDTA) blood samples were collected at the time of operation. Informed consents were obtained from all patients. This study was approved by the institutional review board at our institution (IRB No. 1403-018-562). For normal controls, 195 healthy Koreans were used.

### Genotyping of FoxP3 gene polymorphisms

In 30 patients, genomic DNA was prepared from peripheral blood using a QIAamp DNA Blood Mini Kit (Qiagen, Valencia, CA). Four FoxP3 polymorphisms (rs5902434 del/ATT, rs3761548 C/A, rs3761549 C/T and rs2232365 G/A) were analyzed by PCR-sequencing (Table 1). PCR was performed with a total of 40 uL reaction mixture containing 40 ng DNA, 2 uL of 10 pmole/uL concentration of each primer, 0.8 uL of each 10 mM dNTP mix, 2.0 mM MgCl_2_ and 1.0 U Taq DNA polymerase (Roche applied science, Basel, Switzerland) with 4uL of 10X reaction buffer. Protocol consisted of an initial denaturation step at 95 °C for 5 min; 30 cycles of 30 s denaturation step at 95 °C, 30 s annealing step at each annealing temperature (Table 1) and 30 s extension step at 72 °C, and with a 5 min final extension step at 72 °C.

**Table 1:** Sequencing primers

Polymorphism	Primer	5′-3′	Length (bps)	AT
rs5902434	FP3-1 F	CTGCTCTCCCCTACCAGATG	196 bp	56 °C
del/ATT	FP3-1R	CCCTGCCCATGCATTAAGTA		
rs3761548	FP3-2 F	TTGTCTACTCCACGCCTCTCC	373 bp	60 °C
C/A	FP3-2R	TGCCTCCATCATCACCACG		
rs3761549	FP3-4 F	GTCCTCTCCACAACCCAAGA	250 bp	60 °C
C/T	FP3-4R	CAGATTTTTCCGCCATTGAC		
rs2232365	FP3-3 F	GAGGGCTTTCAAGGTGAGGA	371 bp	60 °C
G/A	FP3-3R	GGGAGTTGGATTGGGTGCA		

*bps* base pairs, *AT* annealing temperature

Five μL of the PCR products were mixed with 2 μL of ExoSAP-IT PCR Clean Up (Affymetrix, Inc., Santa Clara, CA, USA) and were incubated at 37 °C for 15 min and 80 °C for 15 min. Four uL of BigDye Terminator Ready Reaction Mix (Life technologies, Grand Island, NY, USA), 1 uL of 5 pmole/uL sequencing primer, and 4 uL of deionized water were added to 1 uL of purified PCR product with 30 thermal cycles (96 °C for 10 s, 50 °C for 5 s, and 60 °C for 4 min). Two uL of 3 M sodium acetate/EDTA buffer (pH 4.6) and 25 uL of absolute EtOH were added. After vigorous vortexing and centrifugation at 2000 g for 30 min, supernatant was removed. Addition of 50 uL of 80% EtOH and centrifugation at 2000 g for 5 min were repeated twice. Fifteen uL of Hi-Di Formamide (Life technologies, Grand Island, NY, USA) was added and heated for 4 min at 95 °C. Samples were loaded on ABI 3730XL DNA analyzer (Applied Biosystems, Foster City, CA, USA). The electropherograms were processed using Chromas Lite 2.1.1 (Technelysium Pty Ltd., Brisbane, Australia).

### Statistical analysis

The SNPs were selected based on CHB haplotype data using the HAPMAP database (http://www.hapmap.org). Observed genotype frequency was tested by Hardy–Weinberg equilibrium (HWE) on males and females separately because *FoxP3* gene is located on *Xp*11.23. Odds ratio (OR) and confidence interval (CI) were calculated. Frequencies of the genotypes of four FoxP3 polymorphisms in RRP patient and controls were compared by Fisher’s exact test or Chi-square test as appropriate, and also on males and females separately, because *FoxP3* gene maps to X*p*11.23. The level of significance was set at *p* < 0.05, and odds ratios (ORs) with 95% confidence intervals (CI) were calculated for those comparisons showing significant *p* values.

## Results

Genotype frequencies (GF) of FoxP3 polymorphisms in RRP patients and controls are presented in Table 2. GF of FoxP3 polymorphisms in male RRP patients are not different from those in controls. GF of rs5902434 del/ATT were significantly increased in female RRP patients than controls (66.7% vs 43.0%, *p* = 0.043, OR = 3.1 [95% CI, 1.0-9.7]). GF of rs5902434 ATT/ATT were significantly decreased in female RRP patients than controls (0.0% vs 23.0%, *p* = 0.039, OR = 9.4 [95% CI, 1.1-163.1]). GF of rs2232365 AG were significantly increased in female RRP patients than controls (73.3% vs 43.0%, *p* = 0.028, OR = 3.6 [95% CI, 1.1-12.2]). GF of rs2232365 GG was significantly decreased in female RRP patients than controls (0.0% vs 23.0%, 761548, *p* = 0.039, OR = 9.4 [95% CI, 1.1-163.1]). GF of rs3761548 A/C and rs3761549 showed no statistically significant difference between RRP patients and controls.

**Table 2:** Associations of *FOXp3* polymorphisms with RRP

	Total (*n* = 225)			Male (*n* = 110)			Female (*n* = 115)			
	Case (*n* = 30)	Control (*n* = 195)		Case (*n* = 15)	Control (*n* = 95)		Case (*n* = 15)	Control (*n* = 100)		
	n (%)	n (%)	*p*	n (%)	n (%)	*p*	n (%)	n (%)	*p*	OR (95% CI)
Age, mean (range)	41.2 (4–80)	53.0 (41–71)	ns	42.5 (4–80)	55.1 (41–70)	ns	40.3 (7–69)	51.0 (41–71)	ns	
rs5902434										
del/del	15(50.0)	99(50.8)	ns	10(66.7)	65(68.4)	ns	5(33.3)	34(34.0)	ns	
del/ATT	10(33.3)	43(22.1)	ns				10(66.7)	43(43.0)	0.043	3.1 (1.0–9.7)
ATT/ATT	5(16.7)	53(27.2)	ns	5(33.3)	30(31.6)	ns	0(0.0)	23(23.0)	0.039	9.4 (1.1–163.1)
del allele	30(66.7)	176(59.7)	ns	10(66.7)	65(68.4)	ns	20(66.7)	111(55.5)	ns	
rs3761548										
CC	24(80.0)	141(72.3)	ns	12(80.0)	81(85.3)	ns	12(80.0)	60(60.0)	ns	
AC	3(10.0)	34(17.4)	ns				3(20.0)	34(34.0)	ns	
AA	3(10.0)	20(10.3)	ns	3(20.0)	14(14.7)	ns	0(0.0)	6(6.0)	ns	
C allele	39(86.7)	235(79.7)	ns	12(80.0)	81(85.3)	ns	27(90.0)	154(77.0)	ns	
rs3761549										
CC	20(66.7)	141(72.3)	ns	13(86.7)	79(83.2)	ns	7(46.7)	62(62.0)	ns	
CT	8(26.7)	33(16.9)	ns				8(53.3)	33(33.0)	ns	
TT	2(6.7)	21(10.8)	ns	2(13.3)	16(16.8)	ns	0(0.0)	5(5.0)	ns	
T allele	35(77.8)	236(80.0)	ns	13(86.7)	79(83.2)	ns	22(73.3)	157(78.5)	ns	
rs2232365										
AA	14(46.7)	100(51.3)	ns	10(66.7)	66(69.5)	ns	4(26.7)	34(34.0)	ns	
AG	11(36.7)	43(22.1)	ns				11(73.3)	43(43.0)	0.028	3.6 (1.1–12.2)
GG	5(16.7)	52(26.7)	ns	5(33.3)	29(30.5)	ns	0(0.0)	23(23.0)	0.039	9.4 (1.1–163.1)
A allele	29(64.4)	177(60.0)	ns	10(66.7)	66(69.5)	ns	19(63.3)	111(55.5)	ns	

*RRP* recurrent respiratory papillomatosis,OR odds ratio, CI confidence interval

## Discussion

It is still not known why only a very small proportion of HPV-exposed subjects have recurrent respiratory papillomatosis (RRP). The local HPV-specific immune responses that allow chronic HPV-6 and -11 infections in RRP patients was suggested. As a possible mechanism, the dominance of Th2-like cytokine (IL-4 and IL-10) responses and decreased secretion of IFN-γ were reported by peripheral blood mononuclear cells (PBMC) exposed to autologous papilloma tissues in patients with RRP [9]. Recently, impaired function of Tregs was also suggested as a possible mechanism for developing RRP [4].

FoxP3 is crucial regulatory factor for the development and function of Tregs. Polymorphisms have been reported in various regions of the *FOXP3* gene, including the promoter, intron and exon regions. Promoters are relevant to initiating transcription and are therefore might harbor functionally involved polymorphisms [10]. Polymorphisms of *FOXP3* gene promoter may affect the function or quantity of Treg, resulting in various autoimmune diseases. Therefore, *Foxp3* gene promoter polymorphisms were associated with various autoimmune diseases [5] and CMV infection in pediatric allogeneic hematopoietic stem cell transplantation [11].

In our study, rs3761548, which was frequently reported to be associated with various diseases [5] showed no association with RRP patients. However, rs5902434 ATT/ATT genotype and rs2232365 GG genotype were significantly decreased in female RRP patients than controls. The rs5902434 del/del genotype was significantly associated with lupus nephritis in Taiwanese [12], and preeclampsia in Chinese [6], but, was not associated with allergic rhinitis [13] and unexplained recurrent spontaneous abortion [14]. The rs2232365 GG genotype was associated with unexplained recurrent spontaneous abortion [14] in Chinese, but showed no association with psoriasis [15] and Crohn’s disease [16]. Polymorphisms of FOXP3 gene promoter may change the binding specificity of transcription factors and are related to initiating transcription, therefore, may affect the function or quantity of Treg [17], resulting in various autoimmune diseases. The reason why the impacts are various in different disease and ethnic groups should be further elucidated. In our study, the associations of *FoxP3* polymorphisms were observed only in female RRP patients. *FoxP3* gene is located on *Xp*11.23. Lyonization of X-chromosome results in only two phenotypes for each polymorphism, which might affect the pathogenesis of RRP in females, should be also clarified in further studies.

Interestingly, for both rs5902434 and rs2232365, the frequencies of heterozygote were significantly higher in female RRP patients than controls. The unusual associations of heterozygote of *FoxP3* rs3761548 (AC genotype) have been reported in allergic rhinitis [13] and Graves’ disease [18]. The mechanism is not clear yet, but the heterozygosity of *Foxp3* polymorphism itself may have unknown but considerable effects on the pathogenesis of various autoimmune disease and also on RRP.

## Conclusions

In conclusion, polymorphisms of *FoxP3* rs5902434 and rs2232365 were associated with female RRP in Koreans. These results suggest the possible role of *Foxp3* polymorphisms in immune tolerance of HPV infection in RRP pathogenesis. Further studies in larger number of patients including other ethnic groups are needed.

## Abbreviations

EDTA: Ethylenediamine tetraacetic acid
FoxP3: Forkhead box P3
GF: Genotype frequencies
HPV: Human papillomavirus
PBMC: Peripheral blood mononuclear cells
RRP: Recurrent respiratory papillomatosis
Treg: Regulatory T
